# Supplementary material for: The effects of genital myiasis on the diversity of the vaginal microbiota in female Bactrian camels
Source: BMC Vet Res. 2022 Mar 5;18:87. doi: 10.1186/s12917-022-03189-5 (PMC8897907; doi:10.1186/s12917-022-03189-5)
Supplement: Supplementary file 5 — Additional file 5. [file 12917_2022_3189_MOESM5_ESM.zip › MPL201709200_16s_yy/Treat1/css/js_css/jsPDF-master.zip!/jsPDF-master/examples/html2pdf/pdf.html]

display/box/float/clear test


toggle
:   - the way
    - the world ends

      bang

      whimper
    - i grow old
    - pluot?

    > bar maids,

    # sing to me, erbarme dich

This is a nonsensical document, but syntactically valid HTML 4.0. All 100% conformant CSS1 agents should be able to render the document elements above this paragraph **indistinguishably** (to the pixel) from this reference rendering, (except font rasterization and form widgets). All discrepancies should be traceable to CSS1 implementation shortcomings. Once you have finished evaluating this test, you can return to the parent page.
